# Bayesian coestimation of phylogeny and sequence alignment

**DOI:** 10.1186/1471-2105-6-83

**Published:** 2005-04-01

**Authors:** Gerton Lunter, István Miklós, Alexei Drummond, Jens Ledet Jensen, Jotun Hein

**Affiliations:** 1 Department of Statistics, University of Oxford, 1 South Parks Road, Oxford OX1 3TG, UK; 2 MTA-ELTE Theoretical Biology and Ecology Group, Pázmány Péter sétány 1/c 1117 Budapest, Hungary; 3 Department of Zoology, University of Oxford, South Parks Road, Oxford OX1 3PS, UK; 4 Department of Mathematical Sciences, University of Aarhus, Ny Munkegade, Building 530, DK-8000 Aarhus C, Denmark

## Abstract

**Background:**

Two central problems in computational biology are the determination of the alignment and phylogeny of a set of biological sequences. The traditional approach to this problem is to first build a multiple alignment of these sequences, followed by a phylogenetic reconstruction step based on this multiple alignment. However, alignment and phylogenetic inference are fundamentally interdependent, and ignoring this fact leads to biased and overconfident estimations. Whether the main interest be in sequence alignment or phylogeny, a major goal of computational biology is the co-estimation of both.

**Results:**

We developed a fully Bayesian Markov chain Monte Carlo method for coestimating phylogeny and sequence alignment, under the Thorne-Kishino-Felsenstein model of substitution and single nucleotide insertion-deletion (indel) events. In our earlier work, we introduced a novel and efficient algorithm, termed the "indel peeling algorithm", which includes indels as phylogenetically informative evolutionary events, and resembles Felsenstein's peeling algorithm for substitutions on a phylogenetic tree. For a fixed alignment, our extension analytically integrates out both substitution and indel events within a proper statistical model, without the need for data augmentation at internal tree nodes, allowing for efficient sampling of tree topologies and edge lengths. To additionally sample multiple alignments, we here introduce an efficient partial Metropolized independence sampler for alignments, and combine these two algorithms into a fully Bayesian co-estimation procedure for the alignment and phylogeny problem.

Our approach results in estimates for the posterior distribution of evolutionary rate parameters, for the maximum *a-posteriori *(MAP) phylogenetic tree, and for the posterior decoding alignment. Estimates for the evolutionary tree and multiple alignment are augmented with confidence estimates for each node height and alignment column. Our results indicate that the patterns in reliability broadly correspond to structural features of the proteins, and thus provides biologically meaningful information which is not existent in the usual point-estimate of the alignment. Our methods can handle input data of moderate size (10–20 protein sequences, each 100–200 bp), which we analyzed overnight on a standard 2 GHz personal computer.

**Conclusion:**

Joint analysis of multiple sequence alignment, evolutionary trees and additional evolutionary parameters can be now done within a single coherent statistical framework.

## Background

Two central problems in computational biology are the determination of the alignment and phylogeny of a set of biological sequences. Current methods first align the sequences, and then infer the phylogeny given this fixed alignment. Several software packages are available that deal with one or both of these sub-problems. For example, ClustalW [1] and T-Coffee [2] are popular sequence alignment packages, while MrBayes [3], PAUP* [4] and Phylip [5] all provide phylogenetic reconstruction and inference. Despite working very well in practice, these methods share some problems. First, the separation into a multiple-alignment step and a phylogenetic inference step, is fundamentally flawed. The two inference problems are mutually dependent, and alignments and phylogeny should ideally be co-estimated, a point first made by Sankoff, Morel and Cedergren [6]. Indeed, a proper weighting of mutation events in multiple sequences requires a tree, which in turn can only be determined if a multiple alignment is available. For instance, ClustalW and T-Coffee compute their alignments based on a neighbour-joining guide tree, biasing subsequent phylogenetic estimates based on the resulting alignment. Moreover, fixing the alignment after the first step ignores the residual uncertainty in the alignment, resulting in an overconfident phylogenetic estimate.

This leads on to the second issue, which is that heuristic methods are used to deal with insertions and deletions (indels), and sometimes also substitutions. This lack of a proper statistical framework makes it very difficult to accurately assess the reliability of the alignment estimate, and the phylogeny depending on it.

The relevance of statistical approaches to evolutionary inference has long been recognised. Time-continuous Markov models for substitution processes were introduced more than three decades ago [7]. Inference methods based on these have been considerably improved since then [8], and now have all but replaced older parsimony methods for phylogeny reconstruction. With alignments, progress towards statistically grounded methods has been slower. The idea to investigate insertions and deletions in a statistical framework was first considered by Bishop and Thompson [9]. The first evolutionary model, termed the TKF91 model, and corresponding statistical tools for pairwise sequence alignment were published by Thorne, Kishino and Felsenstein [10]. Its extension to multiple sequences related by a tree has been intensively investigated in the last few years [11-17], and has recently also been extended to RNA gene evolution [18]. Current methods for statistical multiple alignment often computationally demanding, and full maximum likelihood approaches are limited to small trees. Markov chain Monte Carlo techniques can extend these methods to practical problem sizes.

Statistical modelling and MCMC approaches have a long history in population genetic analysis. In particular, coalescent approaches to genealogical inference have been very successful, both in maximum likelihood [19,20] and Bayesian MCMC frameworks [21,22]. The MCMC approach is especially promising, as it allows the analysis of large data sets, as well as nontrivial model extensions, see e.g. [23]. Since divergence times in population genetics are small, alignment is generally straightforward, and genealogical inference from a fixed alignment is well-understood [20,24-26]. However, these approaches have difficulty dealing with indels when sequences are hard to align. Indel events are generally treated as missing data [27], which renders them phylogenetically uninformative. This is unfortunate as indel events can be highly informative of the phylogeny, because of their relative rarity compared to substitution events. Statistical models of alignment and phylogeny often refer to missing data. Not all of these can be integrated out analytically (e.g. tree topology), and these are dealt with using Monte Carlo methods. The efficiency of such approaches depend to a great extent on the choice of missing data. In previous approaches to statistical alignment, the sampled missing data were either unobserved sequences at internal nodes [28], or both internal sequences and alignments between nodes [13], or dealt exclusively with pairwise alignments [29,30]. In all cases the underlying tree was fixed. In [31] we published an efficient algorithm for computing the likelihood of a multiple sequence alignment under the TKF91 model, given a fixed underlying tree. The method analytically sums out all missing data (pertaining to the evolutionary history that generated the alignment), eliminating the need for any data augmentation of the tree. This methodology is referred to in the MCMC literature as *Rao-Blackwellization *[32]. As a result, we can treat indels in a statistically consistent manner with no more than a constant multiplicative cost over existing methods that ignore indels.

The only missing ingredient for a full co-estimation procedure is an alignment sampler. Unfortunately, there exists no Gibbs alignment sampler that corresponds to the analytic algorithm referred to above. In this paper we introduce a partial importance sampler to resample alignments, based on a proposal mechanism built on a partial score-based alignment procedure. This type of sampler supports the data format we need for efficient likelihood calculations, while still achieving good mixing in reasonable running time (see Results).

We implemented the likelihood calculator and the alignment sampler in Java, and interfaced them with an existing MCMC kernel for phylogenetics and population genetics [22]. We demonstrate the practicality of our approach on an analysis of 10 globin sequences.

## Results

### Definition of the TKF model

The TKF91 model is a continuous-time reversible Markov model describing the evolution of nucleotide (or amino acid) sequences. It models three of the main processes in sequence evolution, namely *substitutions*, *insertions *and *deletions *of characters, approximating these as single-character processes. A sequence is represented as a string alternatingly consisting of *links *and characters connected by these links. This string both starts and terminates with a link. Insertions and deletions are modeled through a time-continuous birth-death process of links. When a new link is born, its associated character (by convention, its right neighbour) is chosen from the equilibrium distribution of the substitution process. (The original TKF91 model used a simple substitution process, the Felsenstein-81 model [27]. It is straightforward to replace this by more general nucleotide or amino acid substitution models [33].) When a link dies, its associated character dies too. The leftmost link of the sequence has no corresponding character to its left, and is never deleted. For this reason it is called the *immortal link*.

Since subsequences evolve independently, it is sufficient to describe the evolution of a single character-link pair. In a given finite time span, this pair evolves into a finite subsequence of characters and links. Since insertions originate from links, only the first character of this descendant subsequence may be homologous to the original character, while subsequent ones will have been inserted and therefore not be homologous to ancestral characters. The model as applied to pairwise alignments was solved analytically in [10], see also [34]. Conceptually, the model can be trivially extended to trees, but the corresponding algorithms for likelihood calculations have been developed only recently [11,12,14-16].

Because the TKF91 model is time reversible, the root placement does not influence the likelihood, an observation known as Felsenstein's "Pulley Principle" [27]). Although the algorithms we developed are not manifestly invariant under changes in root placement, in fact they are. We have used time reversibility to check correctness of our implementations.

### Computing the likelihood of a homology structure

**Figure 1:**
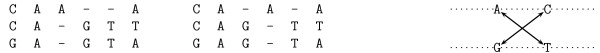
**Alignments and homology structure**. (Left:) Two alignments representing the same homology structure. A "homology structure" is defined as the set of all homology relationships between residues from the given sequences; residues are homologous if they appear in the same alignment column. Our recursion includes contributions from all alignments compatible with a given homology structure (itself represented by a single alignment). (Right:) Due to the evolutionary process acting on the sequences, homology relationships (arrows) will never 'cross' as depicted. This restriction on the equivalence relation ~ is codified by <_*h *_(see text).

The concept of *homology structure *[31], also known as effective alignment [35], refers to an alignment of sequences at leaves without reference to the internal tree structure, and without specifying the ordering of exchangable columns (see below for more details). We derived a linear-time algorithm that computes the likelihood of observing a set of sequences and their homology structure, given a phylogeny and evolutionary parameters, under the TKF91 model [31]. By definition, this likelihood is the sum of the probabilities of all evolutionary scenarios resulting in the observed data. It was previously shown that such evolutionary scenarios can be described as a path in a multiple-HMM ([13,28]), and the likelihood can thus be calculated as the sum of path probabilities over all such paths, in time polynomial in the number of states. However, this straightforward calculation is infeasible for practical-sized biological problems, since the number of states in the HMM grows exponentially with the number of sequences [16]. Since our algorithm does not feature this exponential blow-up of Markov states, we termed it the *one-state recursion*. In contrast to previous approaches [13,28], the one-state recursion relieves us from the need to store missing data at internal tree nodes, allowing us to change the tree topology without having to resample this missing data. This enables us to consider the *tree *as a parameter, and efficiently sample from tree space. The concept of homology structure referred to above is key to our algorithm, and we will presently define this concept more precisely. Let *A*_1_, *A*_2_, ...*A*_*m *_be sequences, related by a tree *T *with vertex set *V*. Let  denote the *j*th character of sequence *A*_*i*_, and let  denote its *k *long prefix. A *homology structure * on *A*_1_, ..., *A*_*m *_is an equivalence relation ~ on the set of all the characters of the sequences, *C *= {}, specifying which characters are homologous to which. The evolutionary indel process generating the homology structure on the sequences imposes constraints on the equivalence relations that may occur. More precisely, the equivalence relation ~ has the property that a total ordering, <_*h*_, exists on *C *such that



(Here, *a *= _*h *_*b *is equivalent to: *a *≮_*h *_*b *and *b *≮_*h *_*a*.) In particular, these conditions imply that the characters constituting a single sequence are mutually nonhomologous. The ordering *<*_*h *_corresponds to the ordering of columns of homologous characters in an alignment. Note that for a given homology structure, this ordering may not be unique (see Fig. 1). This many-to-one relationship of alignment to homology structure is the reason for introducing the concept of homology structure, instead of using the more common concept of alignment.

**Figure 2:**
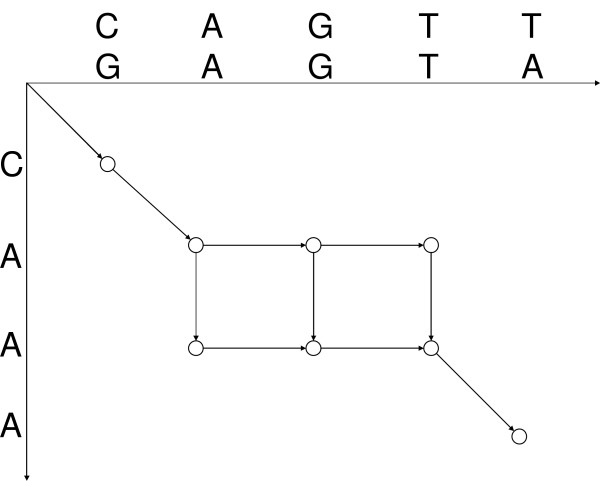
**Dynamic programming table traversal**. The multiple alignment prefixes (represented by o symbols) traversed by the one-state recursion, when the input is the homology structure of Fig. 1. (For clarity, the vectors are plotted in two dimensions instead of the actual three.) The homology structure is represented by the graph, and each directed path on this graph uniquely corresponds to an alignment that is compatible with the homology structure.

The one-state recursion, which calculates the likelihood of a homology structure, is a convolution of two dynamic programming algorithms. The top-level algorithm traverses the prefix set of the multiple alignments representing the homology structure (see Figure 2). This repeatedly calls on a reverse traversal algorithm on the phylogenetic tree, which sums out the likelihood contributions of substitutions and indels under the TKF91 model. See [31] for full details.

### A partial Metropolized independence sampler

Because our algorithm does not require the phylogenetic tree to be augmented with missing data, proposing changes to the evolutionary tree is easy, and mixing in tree space is very good. The drawback however is that without data augmentation, it is unclear how to perform Gibbs sampling of alignments, and we have to resort to other sampling schemes. One straightforward choice would be a standard Metropolis-Hastings procedure with random changes to the alignment, but we expect slow mixing from such an approach. Another general approach is Metropolized independence sampling. Its performance depends on the difference between the proposal distribution and the target distribution, and this will inevitably become appreciable with growing dimension of the problem, as measured by the number and length of the sequences to be aligned. We therefore opted for a *partial Metropolized independence sampler *[36], where we partly defy the "curse of dimensionality" by resampling only a segment of the current alignment. Above increasing the acceptance ratio, this method has the added advantage of being a more efficient proposal scheme, since the time complexity of the algorithm is proportional to the square of the window size, and so leads to an effective increase in mixing per processor cycle. Metzler *et al*. [29] followed a parallel approach, using a partial Gibbs sampler, and showed that this resulted in faster mixing compared to a full Gibbs sampling step. Since the realignment step may change the window length (measured in alignment columns), to have a reversible Markov chain we need all window sizes to have positive proposal probability. We chose a geometric length distribution, but other distributions can be considered equally well.

#### The proposal algorithm

**Figure 3:**
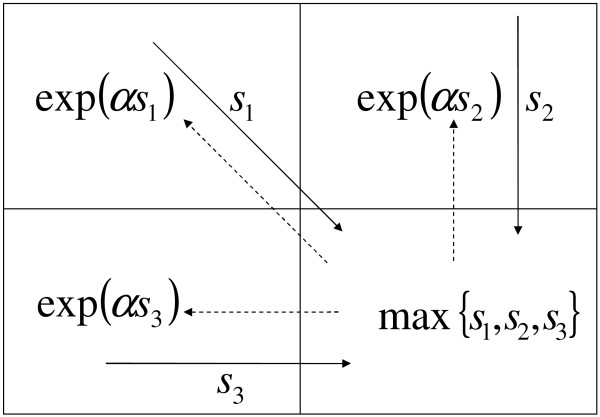
**Generating the proposal alignment**. This figure illustrates the stochastic sequence aligner. In the deterministic fill-in process, the three scores are *s*_1_, *s*_2 _and *s*_3_, hence the value in this cell is max{*s*_1_, *s*_2_, *s*_3_}. In the stochastic traceback phase, the three neighbor cells are choosen with probabilities proportional to exp(*αs*_*i*_), where *α *> 0 is a scaling parameter. The chosen traceback path corresponds to the proposed alignment in the usual way.

The proposal algorithm is as follows. A window size and location is proposed, the alignment of subsequences within this window is removed, and a new alignment is proposed by a stochastic version of the standard score-based progressive pairwise alignment method. First, dynamic programming (DP) tables are filled as for a deterministic score-based multiple alignment, starting at the tree tips and working towards the root, aligning sequences and profiles. We used linear gap penalties, and a similarity scoring matrix that was obtained by taking the log-odds of a probabilistic substitution matrix. The underlying phylogeny was used to define divergence times, and served as alignment guide tree. After filling the DP tables, we applied stochastic traceback. The probabilities for the three possible directions at each position was taken to be proportional to exp(*αs*), where *s *is the deterministic score and *α *is a scale parameter (see Fig. 3). The set of paths that emerged in this way then determined the multiple alignment. All possible alignments can be proposed in this manner, and the proposal as well as the back-proposal probabilities can be calculated straightforwardly.

#### Correctness of the sampler

There are two problems with the proposal sampler introduced above. First, we propose alignments instead of homology structures. We need the latter, since the algorithm derived in this paper calculates the likelihood of the homology structure, not the particular alignment. Although it would be conceptually and (for the sampler) computationally simpler to use alignments, we are not aware of any efficient algorithm that can calculate such *alignment *likelihoods. The second problem is that calculating the proposal probability of a particular alignment is not straightforward. Any choice of window size and location may result in the same proposal alignment. To calculate the true proposal probability of particular alignments, we need to sum over all possible windows, which is prohibitively expensive.

Fortunately, we can solve both problems efficiently. We can sample alignments uniformly inside a homology structure, and at the same time sample homology structures according to their posterior probabilities. As biologically meaningful questions refer to homologies and not particular alignments, it seems reasonable to impose a simple uniform distribution over alignments within homology structures. The second problem is solved by not calculating an alignment proposal probability, but the proposal probability of the combination of an alignment and a resampling window. For a proposal of alignment *X*_2 _and window *w *from a current alignment *X*_1_, we use the following Metropolis-Hastings ratio:



where *H*_1 _and *H*_2 _are homology structures corresponding to the alignments *X*_1 _and *X*_2 _respectively, |*H*_1_| and |*H*_2_| are their cardinalities (i.e. the number of alignments representing these homology structures), and *T *is the proposal probability. Using this ratio, the Markov chain will converge to the desired distribution *π*(*X*) = *π*(*H*)/|*H*|, since the detailed balance condition is satisfied. Indeed,



where the final equality holds because of the symmetry of the left-hand side. The cardinality of a homology structure, |*H*_1_|, is the number of possible directed paths in the graph spanned by the one-state recursion; in other words, the number of permutations of alignment columns that result in alignments compatible with the given homology structure (see Fig. 2). This number can be calculated straightforwardly using a dynamic programming algorithm that traverses the one-state recursion graph [31,37].

## Discussion

**Figure 4:**
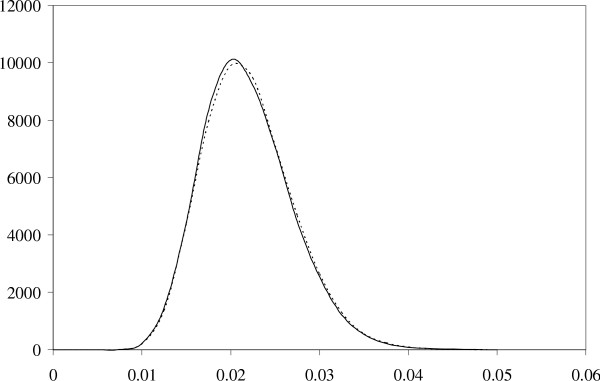
**Posterior distribution of deletion rate *μ***. Estimated posterior densities of the deletion rate *μ *sampled according to *h *(see text), for two independent runs, suggesting excellent convergence. The sampled mean is 0.0207; the 95% highest posterior density (HPD) interval was estimated to be (0.0121, 0.0316).

The one-state recursion provides a method for calculating the likelihood *L *= Pr{*A*, |*T*, *Q*, *λ*, *μ*} of observing the sequences with their homology structure (loosely, "alignment") given the tree and model parameters. Here *A *are the amino acid sequences,  is their homology structure, *T *is the tree including branch lengths, *Q *is the substitution rate matrix, and *λ*, *μ *are the amino acid insertion and deletion rates. To demonstrate the practicality of the new algorithm for likelihood calculation we undertook a Bayesian MCMC analysis of ten globin protein sequences (see Additional file: 1). We chose to use the standard Dayhoff rate matrix to describe the substitution of amino acids. As initial homology structure we used the alignment computed by T-Coffee. We co-estimated homology structures, the parameters of the TKF91 model, and the tree topology and branch lengths. To do this we sampled from the posterior,



where *Z *is the unknown normalising constant. We chose the prior distribution on our parameters, *f *(*T*, *λ*, *μ*), so that *T *was constrained to a molecular clock, and *λ *= *μL*/(*L *+ 1) to make the expected sequence length under the TKF91 model agree with the observed lengths; here *L *is the geometric average sequence length. All other parameters were sampled under uniform priors. We assume a molecular clock to gain insight into the relative divergence times of the alpha-, beta- and myoglobin families. In doing so we incorporate insertion-deletion events as informative events in the evolutionary analysis of the globin family. The posterior density *h *is a complicated function defined on a space of high dimension. We summarise the information it contains by computing the expectations, over *h*, of various statistics of interest. We estimate these expectations by using MCMC to sample from *h*. Marginalizations for continuous variables can be done in a straightforward manner; see for example Figure 4, which depicts the marginal posterior density of the *μ *parameter for two independent MCMC runs, showing excellent convergence.

**Figure 5:**
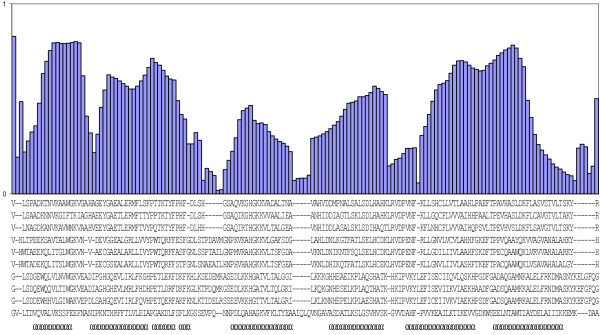
**Maximum posterior decoding alignment, and column reliabilities**. The maximum posterior decoding alignment of ten globins (human, chicken and turtle alpha hemoglobin, beta hemoglobin, myoglobin and bean leghemoglobin). Posterior probabilities for aligned columns were estimated as their rate in the Markov chain. Common alpha helices are indicated with '*α*' symbols under the alignment. A broad correspondence between peaks in the posterior alignment reliability and the position of conserved secondary structure is apparent.

For alignments, the maximum *a-posteriori *alignment is very hard to estimate from an MCMC sample run, as there are typically far too many plausible alignments contributing to the likelihood. Indeed, we found that almost all alignments in a moderately long MCMC run (50000 samples) were unique. However, it is possible to reconstruct a reliable *maximum posterior decoding *[38] alignment from such a moderate long sampling run. This alignment uses the posterior single-column probabilities, which can be estimated much more reliably since many alignments share particular columns, to obtain an alignment that maximizes the product of individual column posteriors. This alignment can be obtained by a simple dynamic programming algorithm [39], see Fig. 5. It is hard to visualise alternative suboptimal alignments, but the individual posterior column probabilities clearly reveal the more and less reliable regions of the alignment. We found that the reliable alignment regions broadly correspond to the alpha helical structure of the globin sequences.

**Figure 6:**
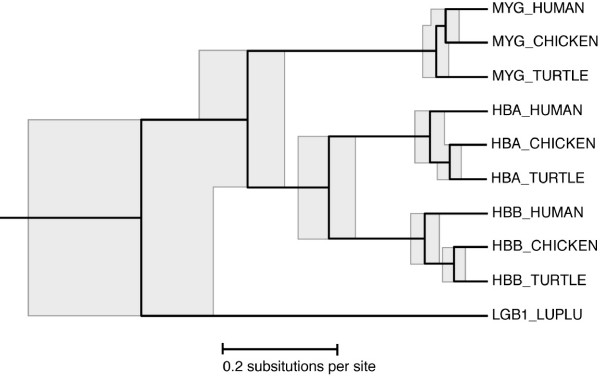
**Maximum *a-posteriori *phylogeny**. The maximum *a posteriori *tree (black) relating the ten globins of Fig. 5, and 95% confidence intervals of the node heights (grey boxes). Most of the tree's topology is well determined, with the exception of the myoglobin sub-tree. Alpha and beta chain sub-families both support the traditional ordering of birds, turtles and mammals, while the three myoglobin sequences support an unconventional phylogeny, as previously observed by Hedges and Poling [41]. However, the posterior probability for the topology of the myoglobin subtree is smaller than that for the remaining topology. The marginal posterior probability (estimated from the MCMC chain) for the monophyly of human and chicken myoglobin is 83.1%, followed by the conventional grouping of turtle and chicken at 11.9%. The third topologlcal arrangement of myoglobin occurred the remaining 5% of the time, suggesting significant homoplasy in this sub-family.

Figure 6 depicts the maximum *a posteriori *(MAP) estimate of the phylogenetic relationships of the sequences. This example exhibits only limited uncertainty in the tree topology, however we observed an increased uncertainty for trees that included divergent sequences, such as bacterial and insect globins (results not shown).

The estimated time of the most recent common ancestor of each of the alpha, beta and myoglobin families are all mutually compatible (result not shown), suggesting that the molecular clock hypothesis is at least approximately valid. Analysis of a four sequence dataset demonstrate consistency in *μ *estimates between MCMC and previous ML analyses [16] (data not shown). Interestingly, the current larger dataset supports a lower value of *μ*. This is probably due to the fact that no indels are apparent within any of the subfamilies despite a considerable sequence divergence. The indel rate estimated by the current cosampling procedure is greater than the estimate on a fixed multiple alignment [31] (0.0207 vs. 0.0187), but this discrepancy is not significant for the current dataset. It should be stressed that the two MCMC analyses of the globin data set presented here are purely illustrative of the practicality of the algorithm described, and no novel biological results were obtained. The two MCMC runs of 5 million states each required less than 12 hours of CPU time each on a 2.0 GHz G5 Apple Macintosh running OS X, using an unoptimised implementation of the algorithm. From these runs we sampled 50000 states each. The estimated number of independent samples (estimated sample size, ESS) for the posterior probabilities was 250 and 240, respectively (see [22] for methods), while for the indel rate *μ *the ESSs were calculated at 5400 and 4000. We expect analyses of data sets of around 50 sequences to be readily attainable with only a few days computation.

## Conclusion

In this paper we present a new cosampling procedure for phylogenetic trees and sequence alignments. The underlying likelihood engine uses recently introduced and highly efficient algorithms based on an evolutionary model (the Thorne-Kishino-Felsenstein model) that combines both the substitution and insertion-deletion processes in a principled way [31]. We show that the proposed method is applicable to medium-sized practical multiple alignment and phylogenetic inference problems.

One motivation for using a fully probabilistic model, and for using a co-estimation procedure for alignments and phylogeny, is that this makes it possible to assess the uncertainties in the inferences. Fixing either the alignment or the phylogeny leads to an underestimate of the uncertainty in the other, and score-based methods give no assessment of uncertainty whatsoever.

We show that the confidence estimates so obtained can contain biologically meaningful information. In the case of the multiple alignment of globin sequences, peaks in the posterior column reliabilities correspond broadly to the various conserved alpha helices that constitute the sequences (see Fig. 5). In the case of the tree estimate, the non-traditional phylogeny supported by the myoglobin subtree coincided with a significant polyphyly, as indicated by the posterior tree topology probabilities, and graphically represented by significantly overlapping 95% node height confidence boxes (see Fig. 6). It is clear that such confidence information significantly contributes to the usefulness of the inference.

At the heart of the method lies a recently introduced algorithm, termed the "indel peeling algorithm", that extends Felsenstein's peeling algorithm to incorporate insertion and deletion events under the TKF91 model [31]. This renders indel events informative for phylogenetic inference. Although incurring considerable algorithmic complications, the resulting algorithm is still linear-time for biological alignments (see also Figure 1). Moreover, our approach allows efficient sampling of tree topologies, as no data is presented at internal nodes.

We also developed a method for sampling multiple alignments, which is applicable for the data augmentation scheme we used for the efficient likelihood calculations. By combining the two samplers, we can co-sample alignments, evolutionary trees and other evolutionary parameters such as indel and substitution rates. The resulting samples from the posterior distribution can be summarized in traditional ways. We obtained maximum *a-posteriori *estimates of alignment, tree and parameters, and augmented these with estimates of reliability.

As was already mentioned in [10], it would be desirable to have a statistical sequence evolution model that deals with 'long' insertions and deletions, instead of single nucleotides at a time. For score-based algorithms, this is analogous to the contrast between linear and affine gap penalties. It is clear that the extension of the model to include long indels would result in considerable improvements, but the algorithmic complexities are considerable. We have made progress on a full likelihood method for statistical sequence alignment under such an evolutionary model [17], but the generalization of this method seems nontrivial. We believe that here too, Markov chain Monte Carlo approaches, combined with data augmentation, will be essential for practical algorithms. However, we also believe that in certain restricted but biologically meaningful situations, such as highly conserved proteins, the TKF91 model is reasonably realistic for the co-estimation procedure presented here to be of practical interest.

## Availability and requirements

The BEAST package (AJ Drummond and A Rambaut), which includes the algorithm described in this paper, is available from , with full installation and requirement details. The data set used in this paper is avaliable (see Additional file: 1)

## Authors' contributions

IM conjectured and GL proved the one-state recursion. GL and IM independently implemented the algorithms, and wrote the paper. JLJ simplified the proof of the recursion, GL suggested to use it within an MCMC phylogeny cosampler, and IM suggested to use a Metropolised importance sampler and proved its correctness. GL and AD interfaced the Java algorithms to the BEAST phylogeny sampling package [40], and AD carried out the MCMC analysis. JH provided project management. All authors read and approved the final manuscript.

## Supplementary Material

Additional File 1 This XML file specifies the MCMC run for the example phylogeny and alignment co-estimation given in this paper (see Figs. 4, 5, 6). To run, download the BEAST package (AJ Drummond and A Rambaut, .) Click here for file
